# Using geo-spatial analysis for assessing the risk of hospital admissions due to community-acquired pneumonia in under-5 children and its association with socially vulnerable areas (Brazil)

**DOI:** 10.1186/s12887-020-02398-x

**Published:** 2020-11-03

**Authors:** Juliana Coelho Pina, Luana Seles Alves, Luiz Henrique Arroyo, Ricardo Alexandre Arcêncio, Ellen Cristina Gondim, Maria Cândida de Carvalho Furtado, Débora Falleiros de Mello

**Affiliations:** 1 Federal University of Santa Catarina, Campus Universitário Reitor João David Ferreira Lima, Trindade, Florianópolis, SC CEP: 88040-900 Brazil; 2 University of São Paulo at Ribeirão Preto College of Nursing, Avenida dos Bandeirantes, 3900, Monte Alegre, Ribeirão Preto, SP CEP: 14040-902 Brazil

**Keywords:** Pneumonia, Child health, Spatial analysis, Primary health care

## Abstract

**Background:**

The concentration of under-5 child morbidity and mortality due to pneumonia in developing countries reflects the social inequities. This study aimed to map and assess the spatial risk for hospitalization due to Community-Acquired Pneumonia in children under 5 years of age and its association with vulnerable areas.

**Methods:**

Ecological study in the city of Ribeirão Preto, state of São Paulo, Brazil. The study population consisted of hospitalized under-5 children, diagnosed with community-acquired pneumonia, in Ribeirão Preto-São Paulo-Brazil, from 2012 to 2013. Data were collected in different databases, by a trained team, between March 2012 and August 2013 and from the 2010 Demographic Census of the Brazilian Institute of Geography and Statistics. The 956 urban census tracts were considered as the units of analysis. The incidence of cases per 10,000 inhabitants was calculated by census tracts during the study period. For the identification of the spatial risk clusters, the Kernel density estimator and the Getis-Ord Gi* technique were performed. Generalized additive models were used to verify the association between areas with social vulnerability and the occurrence of childhood pneumonia.

**Results:**

The study included 265 children under the age of five, hospitalized due to community-acquired pneumonia. A concentration of cases was identified in the regions with greater social vulnerability (low income, poor housing conditions and homelessness), as well as a lower occurrence of cases in the most developed and economically privileged area of the city. The majority of the children lived in territories served by traditional primary healthcare units, in which the health surveillance and family and community focus are limited. It is important to highlight that the tracts with the highest degrees of vulnerability, such as those identified as high vulnerability (urban) and very high vulnerability (subnormal urban clusters).

**Conclusions:**

The results contribute to the comprehension of the social factors involved in child hospitalization due to pneumonia, based on the analysis of the spatial distribution. This approach revealed a strategic tool for diagnosing the disparities as well presenting evidences for the planning in health and strength health care system in achieving equity, welfare and social protection of children.

**Supplementary Information:**

**Supplementary information** accompanies this paper at 10.1186/s12887-020-02398-x.

## Background

The reduction of child deaths from preventable causes is one of the Sustainable Development Goals (SDGs), with targets established for the year 2030 [1]. According to this agenda, one of the main challenges is morbidity and mortality due to pneumonia and, if uncontrolled, in 2030 it is estimated that the disease will be responsible for 735,000 deaths of children under 5 years of age [2]. The actions developed to achieve the Millennium Development Goals, in the period 2000 to 2015, led to a considerable drop in the incidence (30%) and mortality (51%) of pneumonia in young children, globally [3]. However, the most recent world estimate indicates that the disease is still responsible for 8800 deaths of children under 5 years of age per year, with a greater concentration in developing countries [2, 3]. These estimates are alarming, since there are effective interventions for the prevention and management of this disease, available at low cost, such as antibiotics and vaccines [4, 5]. Furthermore, the concentration of child morbidity and mortality from pneumonia in developing countries reflects social inequities, which lead to greater exposure to risk factors and hinder access to preventive, diagnostic and treatment actions for the disease [6].

The issue of social inequities in health refers to another SDG proposed by the United Nations (UN) - the reduction of inequality within and between countries. As Brazil is a country with continental dimensions, it presents marked socioeconomic and cultural differences among its regions, which have an impact on the health of the population, especially among children [7]. Studies in the Brazilian context have suggested a change in risk factors for the worsening of pneumonia among children under 5 years of age, and research is recommended to investigate other risk factors not yet explored, especially in the community [7, 8]. At the community level, there is evidence that pneumonia mainly affects areas where there is a concentration of poverty - such as irregular settlements (known as *favelas*), areas with high environmental pollution and areas of difficult access [2]. Accordingly, pneumonia mainly affects an excluded population, in social and geographic terms [9].

Studies showing the spatial distribution of childhood pneumonia can be found in the literature, both in developed countries [10, 11] and in developing countries [12, 13]. Even in developed countries, cases of agglomeration are observed in more vulnerable areas. It should be noted, however, that there are few studies of spatial analysis in this area. Therefore, although the scientific literature is rich in individual baseline studies, at the aggregate level the evidence for cluster detection is still limited. In order to advance the understanding of the current risk factors for the occurrence and worsening of pneumonia in children, it is considered necessary to identify the individual, collective and institutional aspects involved.

In a Brazilian hospital-based case-control study, we identified, through hierarchical analysis, maternal socioeconomic, reproductive, clinical and care factors involved in the hospitalization of children under 5 years of age for community-acquired pneumonia (CAP), with emphasis on the protective role of primary health care (PHC) [8]. In order to advance the understanding of the dynamics between the factors of different levels involved in the outcome, we proposed a spatial approach to the data of this same study population. Accordingly, the study aimed to map and assess the spatial risk for hospitalization due to Community-Acquired Pneumonia in children under 5 years of age and its association with vulnerable areas in Brazil.

## Method

### Study design and setting

**Fig. 1:**
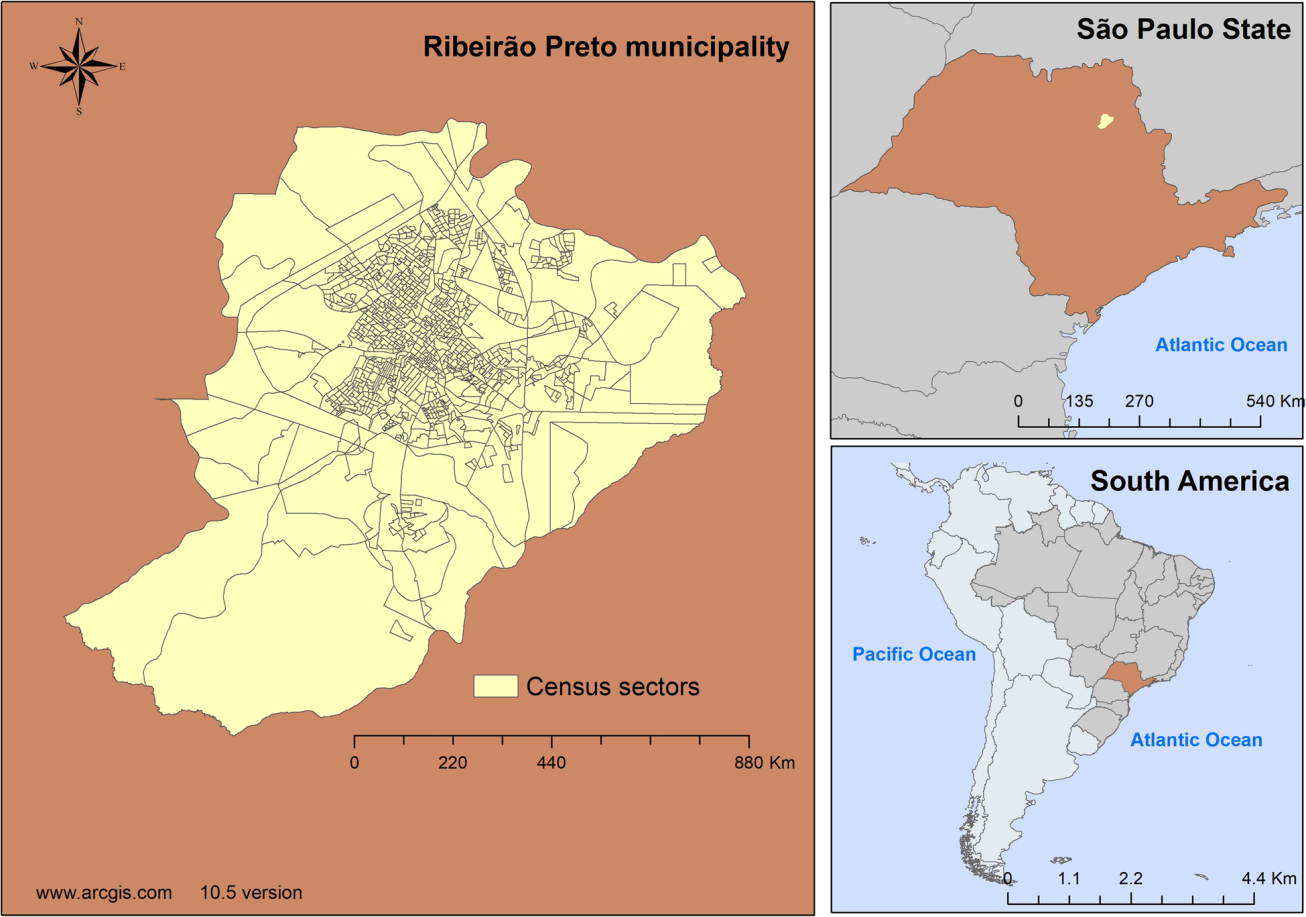
Map of the city studied and its geographical location. Ribeirão Preto-SP, Brazil, 2012–2013. Software used to create the map - ArcGIS 10.5 version. URL link: http://www.arcgis.com. Source: authors

Ecological study [14], carried out in the city of Ribeirão Preto, state of São Paulo (SP), Brazil, located between the geographical coordinates Latitude: 21 ° 10′ 36″ South, Longitude: 47° 49′ 15″ West. The city is in the southeastern region of Brazil, a developing South American country (Fig. 1).

The southeastern region has 43% of the national population and is responsible for 56% of the country’s Gross Domestic Product (GDP). The city of Ribeirão Preto has an area of approximately 650km^2^ and an estimated population of 703,293 inhabitants. The population is mostly urbanized (99.7%) and there is a high demographic density of 928.9 inhabitants per km^2^. It is the city with the tenth largest GDP in the State of São Paulo, being considered an economically important city for the region [15]. Ribeirão Preto is the headquarters of the Regional Health Department XIII (RHDXIII), which is composed of 26 municipalities.

**Fig. 2:**
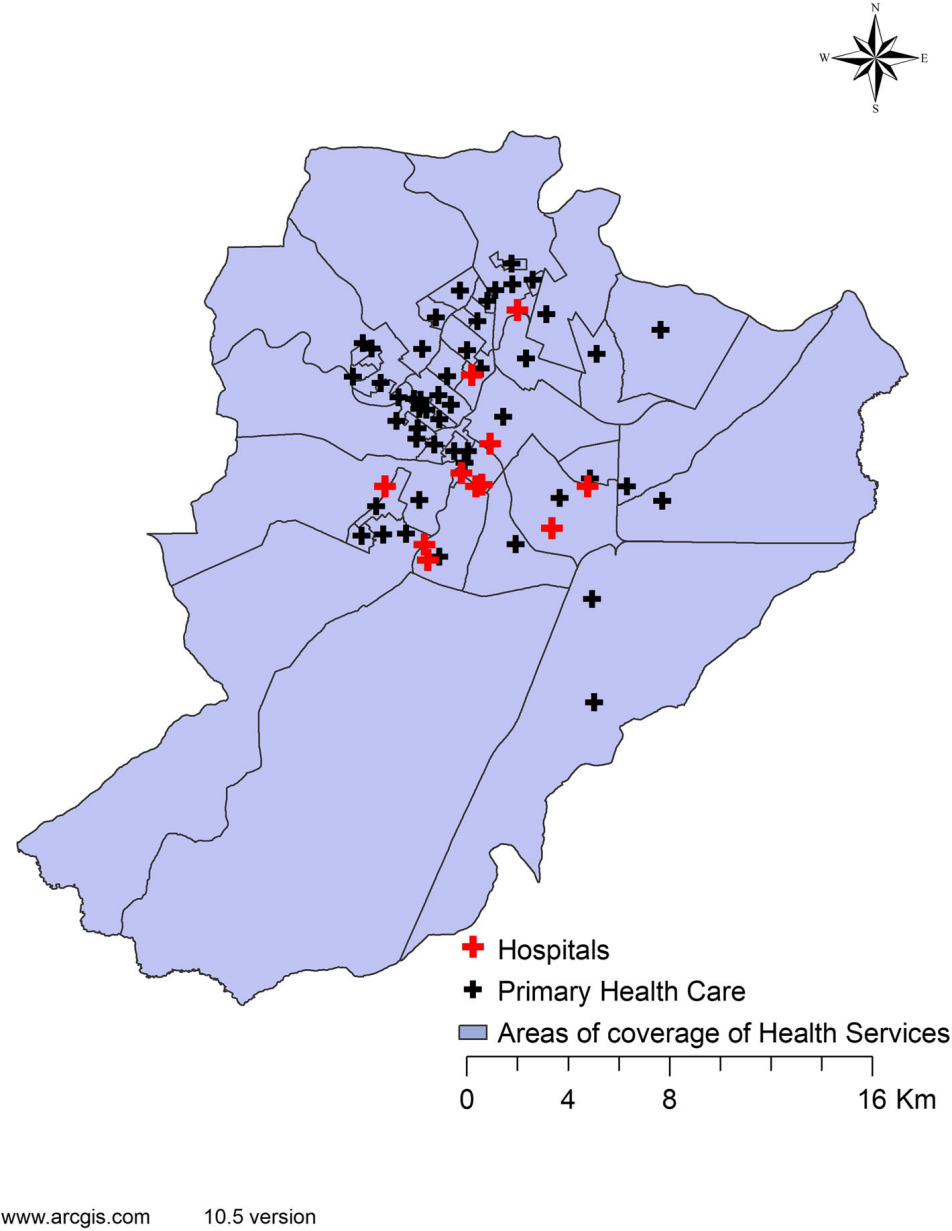
Spatial distribution of health units and respective coverage areas, Ribeirão Preto-SP, Brazil, 2012–2013. Software used to create the map - ArcGIS 10.5 version. URL link: http://www.arcgis.com. Source: authors

The Brazilian public health system is universal and free, called the *Sistema Único de Saúde* (SUS). The Health Care Network of the city studied is divided into five Health Districts: Northern, Southern, Eastern, Western and Central. The health establishments providing public healthcare include 48 PHC units, 13 specialized outpatient clinics, 6 polyclinics, 9 general hospitals and 2 specialized hospitals [16]. Figure 2 illustrates the spatial distribution of the health facilities in the geo-spatial area analyzed.

### Study population and period

The study population consisted of children under the age of five, diagnosed with CAP, hospitalized in three SUS-affiliated hospitals, which are referral centers for pediatric hospitalization in the city and in the health region (RHDXIII). The diagnosis was confirmed by chest radiography and the participants had to be residents in the health region of Ribeirão Preto-SP-Brazil (RHDXIII) and users of the SUS primary health network in the period from 2012 to 2013. Participants who had resided in the location for less than 6 months and those with a recent history of foreign body or liquid aspiration were excluded.

### Source of information and data collection

The data of this study were derived from a hospital-based case-control study [8] and were collected from different databases. A priori, the data were collected by a trained team, between March 2012 and August 2013, in an uninterrupted manner, through daily visits to the participating hospitals. The recruitment of participants followed the order of hospitalization; in the event of refusal or exclusion, the same order was followed for the identification of another eligible child. For the current study, only children living in the urban area of Ribeirão Preto-SP-Brazil were considered. The geographic data were collected from the 2010 Demographic Census of the Brazilian Institute of Geography and Statistics (IBGE) [17].

### Study observation units

For the spatial analysis, the census tract was chosen as an ecological analysis unit, due to the advantage of being the most disaggregated level of population and socioeconomic groups, collected in a systematic, periodic manner and with national standardization [17–19]. The cartographic base of the census tracts in Ribeirão Preto was obtained from the IBGE website, free of charge. For this study, only the urban census tracts in the city were used, corresponding to 956 of the 972 city census tracts.

### Data analysis

Primarily, a database was constructed using the *Excel* 2013 for Windows program, with the characterization data of the participants. For the sociodemographic data, descriptive statistics were performed, with the calculation of measures of absolute frequency and proportions for the categorical variables, using the Statistica software (12.0).

The spatial distribution of cases of hospitalizations due to CAP in children under 5 years of age began with the calculation of the incidence per 10,000 inhabitants by census tracts, during the study period, obtaining an exploratory choropleth map. Next, the search for the geographic coordinates of each address was carried out, using the Google Earth technology, which is accessible without charge, then the technique of georeferencing the cases was performed using the ArcGIS software (v10.5).

For the identification of spatial clusters at risk for the occurrence of CAP hospitalizations in children under 5 years of age, the Radius of the distance was measured using the Incremental Spatial Autocorrelation (ISA) tool provided by the ArcGIS software (10.5), which tested 30 distances, with 7589 km being the result of the most pronounced distance, with *p* < .01 [20]. Subsequently, to verify the spatial association of child hospitalizations for pneumonia, an analysis of the Kernel density estimator and the Getis-Ord Gi* technique was performed. The reason for the application of Kernel is justified by the exploratory approach and, therefore, for the elaboration of maps of point density and the production of a choropleth map. Following this, an analysis of Getis-Ord General G, G(d), was carried out, which consists of a global index to evaluate the spatial association of an attribute based on statistical distances and calculated from the sum of values for a given distance. According to the following formula [19]:

$$ G(d)=\frac{\sum_{i=1}^n{\sum}_{j=1}^n{w}_{ij}(d){x}_i{x}_j}{\sum_{i=1}^n{\sum}_{j=1}^n{x}_i{x}_j},j\  not\ equal\ to\ i $$

The Getis-Ord General G technique is integrated with the Global Moran’s Index, which measures the spatial association of an attribute based on statistical distances and determines the degree of grouping for high and low values [19], when clusters are identified. The null hypothesis of this technique is that there is no spatial grouping. When the *p*-value is significant (*p* < .05), the null hypothesis can be rejected and the z-score value becomes important, as it reflects statistical significance; in this case, if the z-score is positive, the observed G-Index is higher than expected; this indicates that the high values for the attribute are grouped in the study area, configuring a risk value. When the value of the z-score is negative, the observed G-index is lower than the index expected, indicating that low values are grouped in the study area [21]. In addition, the z-score reflects statistical significance, in which +/− 3 have a 99% confidence level [22]. Accordingly, with the intention of examining spatial patterns in detail, the Gi* local association indicator was used. In the Gi*, the values for each location are taken into account, that is, each census tract based on a neighborhood matrix [23].

In order to certify the statistical validity of the results, the pseudo-significance test was used, in which different permutations of the attribute values associated with the regions were generated, where each permutation generated a new spatial arrangement, considering that the values are redistributed across areas. For the analysis of this test, an empirical situation is constructed for the G(d) values. If the value corresponds to the extreme of the simulated distribution, it is an event with significance [24]. For all tests, type I error was set at 5% as statistically significant (*p* < .05). Subsequently, the Getis-Ord Gi* technique was applied, a local association indicator that considers the values for each location - in this case, each census tract - based on a neighborhood matrix. In the analysis, a z-score is generated; for statistically significant census tracts, there is a positive z-score, that is, higher z-scores equate to more intense clusters of high values (hotspots). For negative z-scores, lower z-scores equate to more intense low value groupings (coldspots).

In addition to the z-score, the *p*-value and level of significance (*Gi-Bin*) was provided. The *Gi-Bin* values identify statistically significant clusters, with higher risks being mapped in red and lower risks in blue. Values ranging from + 3 to − 3 reflect statistical significance with a 99% confidence level; + 2 to − 2 a 95% confidence level; + 1 to − 1 a 90% confidence level and, finally, zero indicates no statistical significance [21].

Generalized additive models for location, scale and shape (GAMLSS) [25] were used to verify the association between areas of social vulnerability and the incidence of cases of childhood pneumonia. The use of GAMLSS resulted from their analytical flexibility, considering their ability to adjust to data with overdispersion or that require heterogeneity, allowing for more complex analyses considering the observed data set. Accordingly, the choice of the best model was made considering the lowest Akaike Information Criterion (AIC) value, taking the model that only counted the intercept as a starting point [26].

For the analyses, the number of child hospitalizations for CAP previously presented in the units under analysis was considered as a dependent variable, that is, the urban census tracts in the municipality. The counting of cases per tract took place through the geocoding process of the residential addresses of those affected by the disease. The expected number of cases in the tracts was also incorporated into the GAMLSS model, and for this calculation, the number of total cases and the population of children less than 5 years of age in the municipality were considered as the reference. The expected number of cases is inserted as “offset” and has the function of standardizing the number of cases considering the population residing in the census tracts, enabling a better comparison of cases among the units analyzed [27].

Social vulnerability was investigated through the *Acervo do Índice Paulista de Vulnerabilidade Social* (IPVS –São Paulo Index of Social Vulnerability Collection) prepared by the *Fundação Sistema Estadual de Análise de Dados* (SEADE - State System of Data Analysis Foundation) and based on information derived from the Brazilian Demographic Census of 2010 [17]. This index takes into account variables such as per capita household income, the percentage of women aged 10 to 29 years responsible for the households and the situation of a subnormal cluster (*favela*) in the census tract [28].

The IPVS classifies census tracts into six groups of social vulnerability: Group 1 - lowest vulnerability; Group 2 - very low vulnerability; Group 3 - low vulnerability; Group 4 - medium vulnerability; Group 5 - high vulnerability and Group 6 - very high vulnerability [28]. It should be highlighted that group 6 includes only census tracts classified in the Demographic Census as subnormal clusters, that is, with a very concentrated young and low-income population [17].

The construction of the IPVS is based on a factor analysis that considers different variables representative of the population’s socioeconomic and demographic conditions [28]. It should be noted that in the municipality under analysis a total of 65 census tracts (6.58% of the total urban census tracts) were not properly classified in relation to their social vulnerability, being therefore considered “missing data” and inserted in the model as a factor. Therefore, the IPVS was added to the statistical model as a categorical variable with 7 factors, with group 1 of the lowest social vulnerability being the reference value.

The diagnosis of the final model was carried out, detecting a possible violation of the standard regression premises, that is, verifying the normality of the residuals through the One-sample Kolmogorov-Smirnov test, residuals with zero mean and their homoscedasticity.

### Ethics approval and consent to participate

The study was approved by the Research Ethics Committee of the University of São Paulo at Ribeirão Preto College of Nursing (date: 11/29/2011, authorization number: 1404/2011). Following the recommendations of the Declaration of Helsinki and Resolution 196/96 of the National Health Council, data collection was authorized by hospitals and consent to participate was obtained from a parent or guardian on behalf of the child, by signing a consent form. This consent form signed by parent or guardian’s participants includes authorization for publication in vehicles of scientific dissemination, guaranteeing anonymity.

## Results

**Fig. 3:**
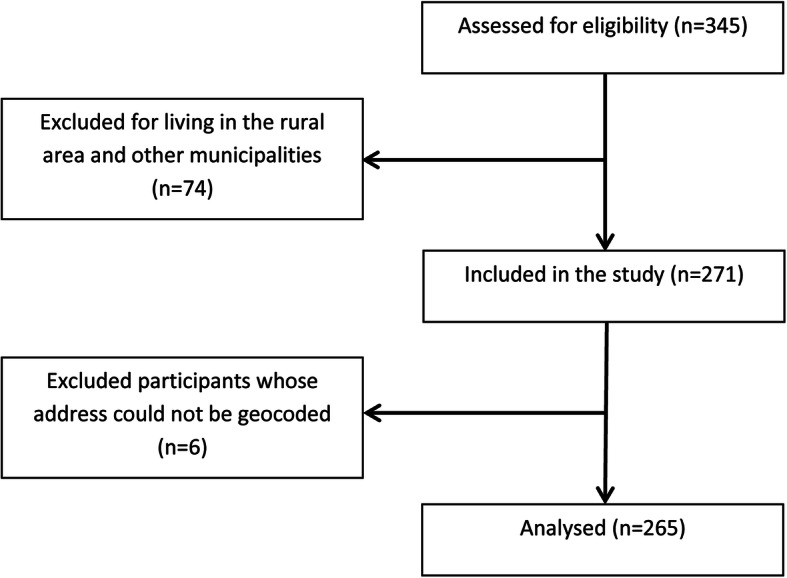
Study flowchart according to STROBE statement, Ribeirão Preto-SP, Brazil, 2012–2013. URL link for STROBE guidelines: http://www.strobe-statement.org. Source: authors

Participants of the study were 265 children hospitalized for CAP, excluding those from the rural area and other municipalities of the RHDXIII (*n* = 74). Furthermore, participants whose address could not be geocoded (*n* = 6) were excluded. The study flow chart is presented in Fig. 3.

The sociodemographic characteristics of the studied population are presented in Table 1.

**Table 1:** Distribution of CAP hospitalization, according to sociodemographic characteristics, Ribeirão Preto-SP, Brazil, 2012–2013

Characteristics	***N***	%
**Sex**		
Male	135	51.0
Female	130	49.0
**Age group (months)**		
02–05.9	60	22.6
06–11.9	66	24.9
12–23.9	76	28.7
24–59.9	63	23.8
**Mother’s age group (years)**		
< 20	20	7.6
20–34.9	197	74.3
≥ 35	48	18.1
**Mother’s schooling (years)**		
0–4	22	8.3
5–8	99	37.4
≥ 9	144	54.3
**Father’s schooling (years)**^a^		
0–4	32	13.5
-8	102	43.0
≥ 9	103	43.5
**Family income - thirds (US$)**^b^		
≤ 931.37	103	38.9
> 931.37 and ≤ 1472.35	80	30.2
> 1472.35	82	30.9

^a^loss of 28 cases in which the interviewee gave no information

^b^value of the US dollar on 12/28/2012 (midpoint of the collection period): 2.04

**Source:** authors

There was an almost homogeneous distribution in relation to sex and age, with a slight concentration of cases in the second year of life. Most of the mothers were young, under 35 years of age, and had studied for more than 9 years. The fathers’ education was slightly lower than that of the mothers - the mean years of study corroborates the categorical data in the table (paternal: 8.1 x maternal 8.8). Regarding family income, there was a concentration in the first third of the distribution (lower income). In addition to the data presented in the table, it should be noted that the mean per capita income was US$293.51, due to the household density observed among the cases (mean of five people in the house, ranging from two to 13 people).

From the address of the children and the information related to the health units, on the website of the Municipal Health Department of Ribeirão Preto-SP, it was possible to identify the PHC unit of reference for the territory, in 254 cases, with the following distribution: 70 (27.5%) cases in areas assigned to Family Health Units (FHUs), 36 (14.2%) had the Primary Health Units (PHUs) as a reference for care with a Community Health Agent Program (CHAP), 127 (50%) with traditional PHUs and 21 (8.3%) cases in areas of the Primary and District Health Units (PDHUs).

Figure 3 shows the health districts with the highest rate of hospitalizations for CAP in children under 5 years of age. The central district presented the census tract with the highest incidence rate - 20,000 cases per 10,000 inhabitants, followed by the western district - with 2000 per 10,000 inhabitants and the northern district - with 133 cases per 10,000 inhabitants.

**Fig. 4:**
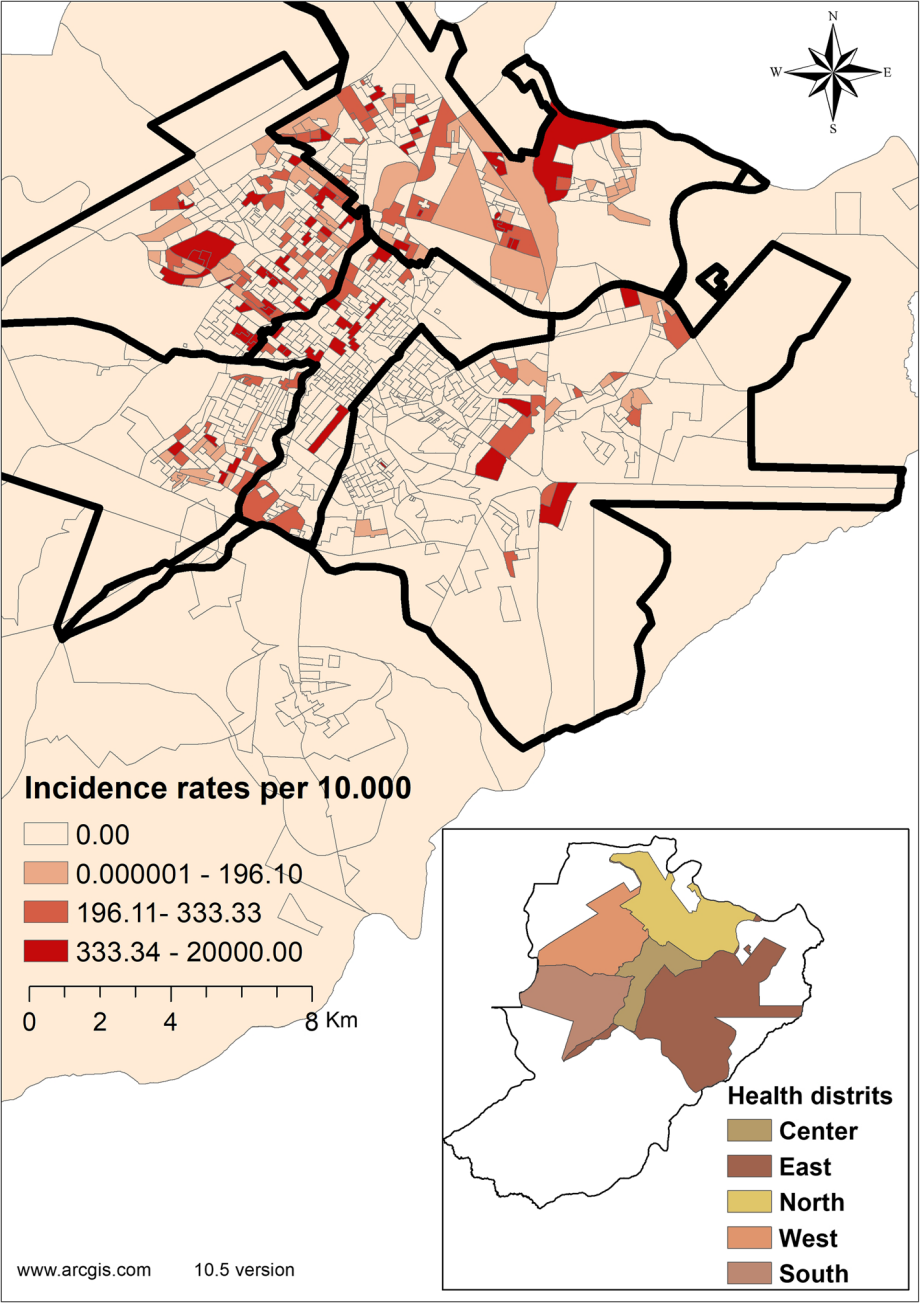
Spatial distribution of CAP Hospitalization. Ribeirão Preto-SP, Brazil, 2012–2013. Software used to create the map - ArcGIS 10.5 version. URL link: http://www.arcgis.com. Source: authors

Figure 4 shows the spatial density of cases of children hospitalized due to CAP, using the Kernel density estimator. When only the density of points is considered, a greater concentration can be observed in the northern and western health districts of the city.

**Fig. 5:**
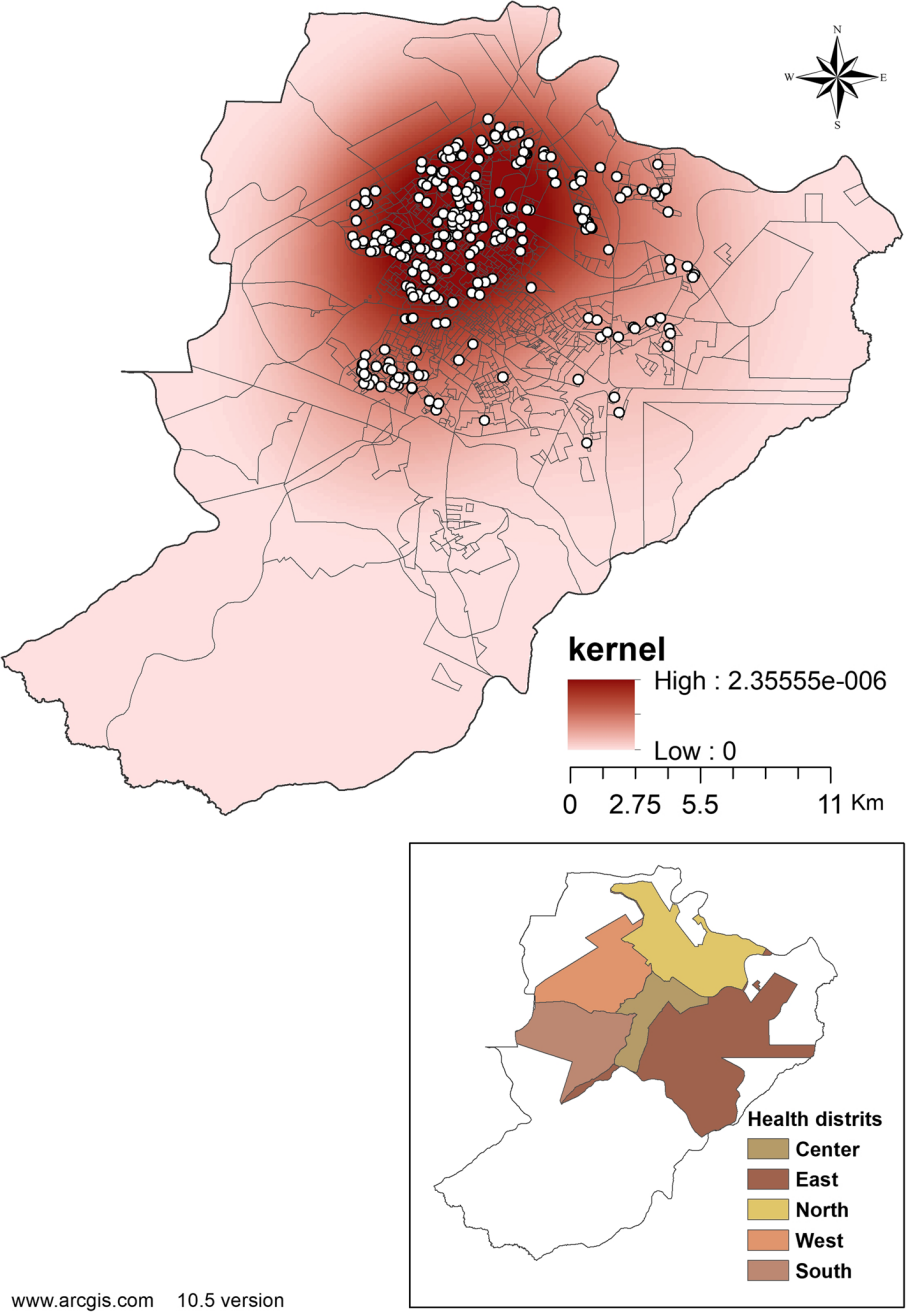
Map of Kernel density of CAP hospitalization. Ribeirão Preto-SP, Brazil, 2012–2013. Software used to create the map - ArcGIS 10.5 version. URL link: http://www.arcgis.com. Source: authors

Regarding the spatial association in Fig. 5, the Getis-Ord General was 0.010, showing a positive spatial relationship, highlighting risk clusters that were also located in the northern and western districts, represented in red - with more intense colors indicating more significant clusters.

**Fig. 6:**
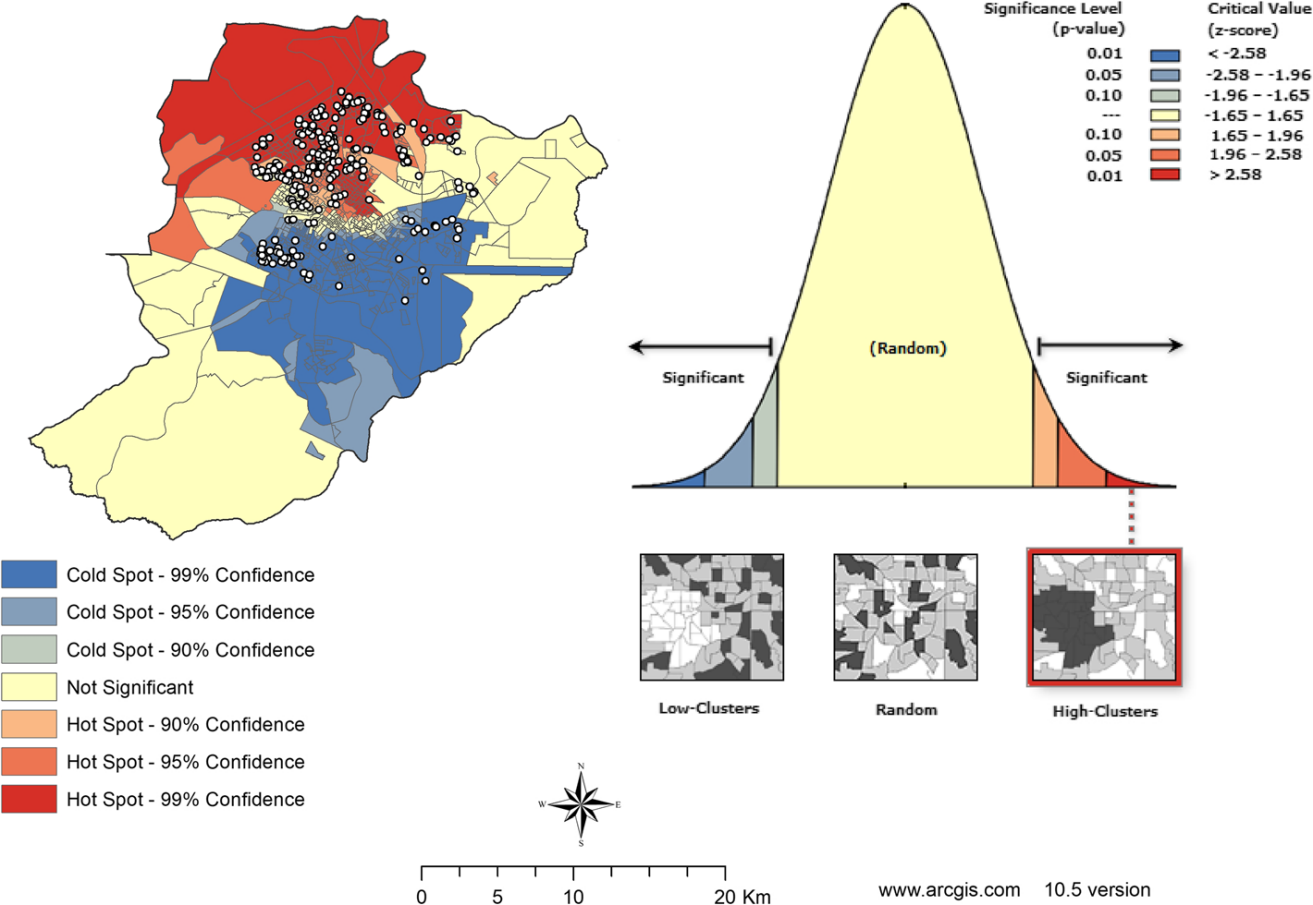
Hotspots and coldspots related to CAP hospitalizations, according to the Gi*. Ribeirão Preto-SP, Brazil, 2012–2013. Software used to create the map - ArcGIS 10.5 version. URL link: http://www.arcgis.com. Source: authors

In contrast, the coldspots*,* shown in blue on the map, also known as protection areas for CAP hospitalizations in children under 5 years of age, were located in the southern and central regions of the city. The variation in the level of significance of the clusters can be observed, with these areas being highly significant (99%CI) (Fig. 6).

Both methods revealed hotspots (high-clusters) in the western, central and southern districts of Ribeirão Preto-SP, with confidence levels of 90, 95 and 99%. In addition, the Getis-Ord Gi* technique identified coldspots (low-clusters) in the eastern district of the city, with confidence levels of 90, 95 and 99%.

The explanatory model developed through the GAMLSS and using the Waring distribution (lowest AIC value of the null model = 1301) made it possible to identify the relationship between the child hospitalizations for CAP and the IPVS. Taking the census tracts with the lowest vulnerability as the reference variable, all other vulnerability classifications had positive coefficients and *p*-values less than 0.05. The positivity of the coefficients can be interpreted as a direct relation to the increase in cases given the increasing vulnerability in the census tracts (Table 2).

**Table 2:** Explanatory model of the relationship between the IPVS and CAP hospitalization, Ribeirão Preto-SP, Brazil, 2012–2013

São Paulo Social Vulnerability Index - IPVS	Coefficient	***P*** value
Group 1 - Lowest vulnerability	–	–
Group 2 - Very low vulnerability	1.6731	<.01
Group 3 - Low vulnerability	1.9576	<.01
Group 4 - Medium vulnerability	2.1685	<.01
Group 5 - High vulnerability (Urban)	2.5457	<.01
Group 6 - Very high vulnerability (subnormal urban clusters)	2.3304	<.01
Not classified	0.3474	.598

**AIC value** 1203.821

**One-sample Kolmogorov-Smirnov test:** *D* = 0.045157, *p*-value = 0.03557

**Source:** authors

It is important to highlight that the tracts with the highest degrees of vulnerability, such as those identified as high vulnerability (urban) and very high vulnerability (subnormal urban clusters), presented the highest coefficient values in the analyses, which can be understood as a greater impact on the mean number cases in the municipality analyzed.

In the diagnosis of the statistical model, the residuals presented normality, seen through the One-sample Kolmogorov-Smirnov test. In addition, the randomized residual quantiles showed a mean of − 0.06 and a variance of 1.05, indicating a good fit of the model to the observed data (see Additional file 1).

## Discussion

The study aimed to map and assess the spatial risk for hospitalization due to Community-Acquired Pneumonia in children less than 5 years of age and its association with vulnerable areas. The findings evidenced geo-spatial locations with higher risk for hospital admissions due to Community-Acquired Pneumonia in children, related to social vulnerability and inequity in these areas, as well as to the difficulty for Primary Health Care to monitor these children.

The central district comprises the oldest region and the commercial center of the city, with considerably less area than the other districts. The estimated population at the time of data collection was 105,246 inhabitants, with a concentrated population and many homeless people in the area (with a basic profile of begging, drug addiction and drunkenness) [29–31]. The predominant economic classes, according to the Brazilian Economic Classification Criteria (CCEB)^1^, were B2 and C1 [31]. In the health network, no FHU was implemented - the population had only one PDHU, three PHUs and a specialized center [32].

The southern district also did not have an FHU at the time of data collection, the health network being composed of one PDHU and three PHUs [33]. This district had an estimated population of 91,372 inhabitants, home to the largest irregular settlement (subnormal urban cluster) and the largest number of families belonging to economic classes D and E in the city, as well as the highest percentage of families without income [30, 31, 33].

There was also a large number of irregular settlements (subnormal urban clusters) in the western district, as well as a high density households; the estimated population was 162,440 inhabitants and economic classes C1 and B2 predominated [31, 33]. However, it was the district that had the most complex health network in the city, consisting of one PDHU, six PHUs, a maternal and child health center, ten FHUs and a specialized clinic [32].

The spatial analysis also identified coldspots in the eastern district - the most prosperous region of the city, with the highest percentage of people belonging to economic classes A1, A2 and B1, as well as the lowest percentage of the population without income [31]. In addition, there was an expansion of real estate speculation in the area, with a higher concentration of high-end residential condominiums [31]. The district had an estimated population of 171,661 inhabitants, being the most populous, however, with a considerably larger geo-spatial area than the other districts, which gave it a low population density [29–31]. Regarding the health network, the district included one PDHU, five PHUs and one FHU [32].

In summary, the spatial analysis revealed a concentration of cases of children hospitalized due to CAP in the regions with greater social vulnerability (low income, poor housing conditions and homelessness), as well as a lower occurrence of cases in the most developed and economically privileged area of the city. Furthermore, it should be highlighted that the explanatory model developed revealed that child hospitalizations due to CAP were associated with the social vulnerability of the population living in the municipality.

Spatial studies developed by other authors have also shown concentrations of cases of childhood pneumonia in areas with lower socioeconomic status, in countries such as Australia [13], Korea [10], United States of America [34], England [35] and Africa [12]. In addition, in the Philippines and England there was a concentration of severe cases of pneumonia in children living in areas further away from the regional referral hospital [35, 36].

The socioeconomic level has been described as an important risk factor for morbidity and mortality due to pneumonia and other lower respiratory infections among children of developing countries [5, 37]. In the present study, although satisfactory maternal age and parental education were observed among the children hospitalized for CAP, the majority belonged to families with per capita income (US$293.51) below the national (US$515.69), state (US$701.96) and municipal (US$644.14) for the period [38].

In the scientific literature, the effects of social inequities on the occurrence and severity of pneumonia have been discussed, especially among children under 5 years of age [2, 6]. Differences in disease morbidity and mortality have been found among populations from different social strata, with severe pneumonia in young children being considered a reflection of poverty [2, 6].

Brazil has marked socioeconomic and cultural differences among its different regions. Furthermore, within the same state or city there are also marked disparities, with children whose family socioeconomic pattern is compatible with highly developed regions and children in situations of poverty. Studies developed in São Paulo, considered the most prosperous state of the country, indicated the highest occurrence of pneumonia among children whose socioeconomic condition is similar to that of children living in the states of the northeastern region, among the poorest in Brazil [7, 8].

The effect of socioeconomic status on child hospitalization for pneumonia is mediated by issues such as maternal age and education, as well as access to resources [37]. However, it is possible that this effect is also mediated by other factors, since the evidence related to the impact of these variables on the severity of childhood pneumonia varies according to the context studied. In the present study, the age and education of the majority of the mothers was satisfactory, although the income was low; an effect of income on the outcome was also observed in the case-control study from which the data of this research are derived [8], regardless of parents’ education levels.

From the spatial analysis of the present study, the results confirmed that the distribution of pneumonia in the geo-spatial area was coincident with greater socially vulnerable areas and poverty. Accordingly, low-income populations occupy marginalized spaces, lacking access to resources and services [9, 39], with this situation constituting a social vulnerability, which could possibly even overcome individual and family protective factors. For example, a family with a well-nourished child, whose parents have a good level of education, but live in an area of social vulnerability (such as subnormal urban clusters or *favelas*) due to low income, may have difficulty taking the child to the health service at the first signs of the disease, causing the evolution of a severe condition, which requires hospitalization.

As pneumonia affects mainly impoverished areas and *favelas* or those with high social vulnerability and poverty, the population at greatest risk has a limited potential for political mobilization. Furthermore, the disease is not easily transmitted across social boundaries, unlike other infectious diseases, which also pose a risk to more developed countries and wealthier areas. In this context, childhood pneumonia continues to be a socially contained and politically neglected disease [2].

Accordingly, the global mobilization for the control of childhood pneumonia is a key point, requiring a broader understanding of the dynamics between individual and collective factors. Interventions aimed at strengthening individual protection, for example, may have different impacts according to the situation and the social space to which the child belongs.

The results of a study that compared clinical research on the effectiveness of the pneumococcal vaccine in children from different countries illustrated this dynamic, with differences found in the protective effect of the intervention, according to the country’s level of development and differences also observed within the same country, according to the area of residence [40]. The hypothesis suggested is that, in areas with adequate access to health services, children are diagnosed at the first signs of respiratory impairment and receive early treatment, decreasing the magnitude of the vaccine’s protective effect; whereas, in areas with problems of access to early diagnosis and treatment, the protective effect of the vaccine on the child population would have a greater impact. Furthermore, in addition to protection at the individual level, vaccination coverage of the exposed population contributes to collective protection, controlling the circulation of the etiological agent [40].

The non-governmental organization Save the Children advocates the development of action plans for pneumonia, integrated with development strategies for the health systems [2]. The plans must involve the investigation of children in areas of higher risk, ensuring access to trained teams, equipped for the proper diagnosis and treatment of the disease and supported by an effective referral system [2].

The Brazilian health system is guided by the organization in Health Care Networks, with PHC being the main gateway, care coordinator and organizer of the actions and services available in the network, aiming to optimize access and the use of the existing resources [41]. The relevance of integrating child monitoring services and effective referral and counter referral mechanisms should be noted [42], particularly in the first years of life, when vulnerabilities to morbidities are greater.

The case-control study from which the sample of this research was derived identified, through hierarchical analysis, that eight of the ten variables that composed the final model referred to actions developed at the PHC level, especially those related to the monitoring and vaccination of the child, prenatal care and family planning. Furthermore, the quality of the PHC itself, assessed from the perspective of the child’s caregiver, had an important protective effect on the hospitalization of young children due to CAP [8].

The results of the present study indicate that the majority of children hospitalized for CAP lived in territories served by traditional PHC Units, called *unidades básicas de saúde* - UBS in Brazil. These units offer appointment and walk-in care in the dentistry, clinical medical, pediatric, gynecology and obstetric areas, as well as vaccination and pharmaceutical assistance. However, in this care model there is no family and community focus, which are attributes of PHC and are provided by the Family Health Strategy (FHS) and CHAP. The monitoring of families over time, as well as the home and community approach, allows the social factors involved in the health-disease process to be identified and understood [43].

Ensuring access to health services is one of the priorities in a plan to control childhood pneumonia. However, it should be noted that the physical presence of the health unit in a given territory does not guarantee access, since it presupposes a bond, extended opening hours, active searches in the community, ease in scheduling appointments and meeting the spontaneous demand, in addition to absence of geographical and cultural barriers, among other aspects [44]. A PHC model that seeks to overcome social inequalities must have a family and community orientation, in order to understand its territory and population and prevent them from becoming health inequities.

Considering that the morbidity and mortality due to preventable diseases and health inequities affect the universal rights of the child, it is understood that the results of this study strengthen the perspective of the performance of health services in the defense of children’s rights.

The study provides evidence of the critical areas in relation to community-acquired pneumonia in children and its association with situations of social vulnerability and poverty, which may contribute to the strengthening of the Health Care Network and support public policies to reduce child mortality, for the Sustainable Development Goals and Agenda 2030. For the future, new investigations could verify whether there were changes in the epidemiological reality identified in the study and whether, with the context of the COVID-19 pandemic, there was cross infection with community pneumonia and how severely this has occurred in children. It would also be opportune to follow these children with a view to observing recurrences and/or new episodes due to reinfection. Considering the recent changes in the political and epidemiological scenarios, it is understood that the replication of these analyses may provide support for the discussion of the impact of the social context on the different etiologies of severe respiratory diseases in childhood.

As a limitation, it is emphasized that this study was carried out in a complementary way to the multivariate analysis, performed in a previous study, in which the other factors involved in child hospitalization for CAP were evaluated, with the data being aggregated in this study. Another limitation is related to ecological fallacy, in which the association observed in the study, does not necessarily hold for the individual level. Another issue is the time, the data was collected 7 years ago, however, the results are current and relevant for understanding the spatial dynamics of the Hospital Admissions due to CAP in Children in the geo-spatial area and for assessing whether the local policies and strategic actions have been addressed to modify the reality revealed in the study.

## Conclusion

The present study identified the concentration of cases of hospitalization of children due to CAP in regions of greater social vulnerability and in areas covered by traditional PHC units, in which health surveillance and the family and community focus were limited. The results contribute to broaden the comprehension of the social factors involved in the hospitalization of children under 5 years of age due to CAP, based on spatial distribution analysis, and address their interface with individual and institutional factors. It can be concluded that knowing and analyzing the geographic area and the characteristics of the registered population provides tools for PHC services to guarantee the access of the most vulnerable children to effective interventions for the control of CAP.

## Supplementary Information

**Additional file 1.** Diagnosis of the statistical model to social vulnerability and CAP hospitalization, Ribeirão Preto-SP, Brazil, 2012–2013. Diagnosis of the statistical model elaborated through the Generalized additive models for location, scale and shape.

## Data Availability

The geographic datasets analyzed in the study are available from the Brazilian Institute of Geography and Statistics repository, available at: <http://www.ibge.gov.br/home/estatistica/populacao/censo2010/resultados_gerais_amostra/default_resultados_gerais_amostra.shtm>. Data of social vulnerability was investigated through the Acervo do Índice Paulista de Vulnerabilidade Social (IPVS –São Paulo Index of Social Vulnerability Collection), available at: <http://ipvs.seade.gov.br/>. The other datasets analyzed in the study are not publicly available due to the guarantee of the participants’ anonymity (they are composed of residential addresses and other personal information), however, are available from the corresponding author upon reasonable request.

## Abbreviations

SDGs: Sustainable Development Goals
UN: United Nations
CAP: Community-acquired pneumonia
PHC: Primary Health Care
GDP: Gross Domestic Product
RHDXIII: Regional Health Department XIII
SUS: Brazilian Nation Health System (*Sistema Único de Saúde*)
SP: State of São Paulo
IBGE: Brazilian Institute of Geography and Statistics
ISA: Incremental Spatial Autocorrelation
GAMLSS: Generalized additive models for location, scale and shape
AIC: Akaike Information Criterion
IPVS: Índice Paulista de Vulnerabilidade Social
SEADE: Fundação Sistema Estadual de Análise de Dados
FHU: Family Health Unit
PHU: Primary Health Unit
CHAP: Community Health Agents Program
PDHU: Primary and District Health Unit
CCEB: Brazilian Economic Classification Criteria (*Critérios de Classificação Econômica do Brasil*)
FHS: Family Health Strategy

## Footnotes

1 The methodology to define the CCEB classes uses a sum of points attributed to the possession of items and the level of education of the head of the family, generating 7 categories, among which A1 represents the highest purchasing power and E represents the most disadvantaged.
