# Supplementary figures and images for: Using geo-spatial analysis for assessing the risk of hospital admissions due to community-acquired pneumonia in under-5 children and its association with socially vulnerable areas (Brazil)
Source: BMC Pediatr. 2020 Nov 3;20:502. doi: 10.1186/s12887-020-02398-x (PMC7606062; doi:10.1186/s12887-020-02398-x)

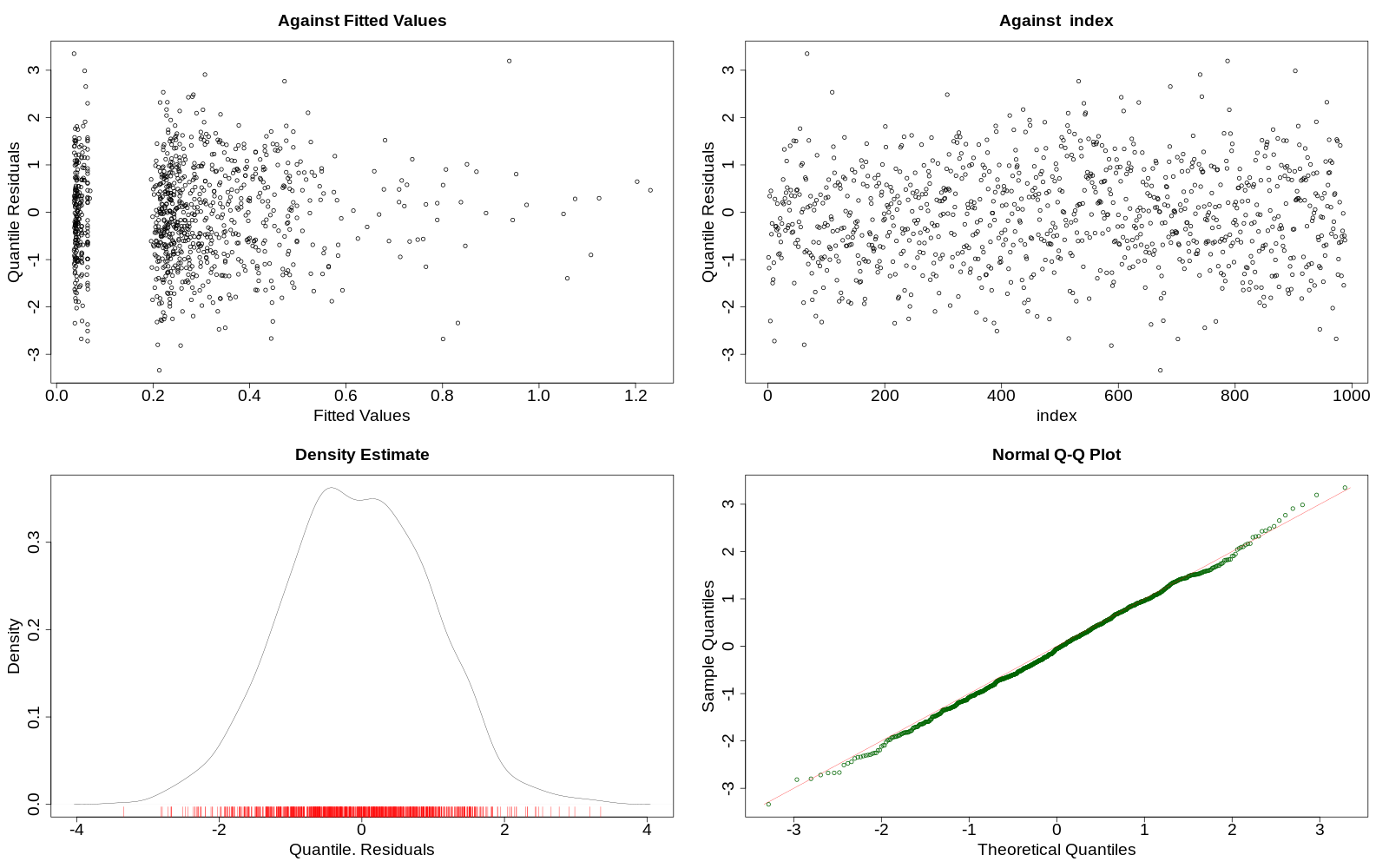

Supplement: Supplementary file 1 — Additional file 1. Diagnosis of the statistical model to social vulnerability and CAP hospitalization, Ribeirão Preto-SP, Brazil, 2012–2013. Diagnosis of the statistical model elaborated through the Generalized additive models for location, scale and shape. [file 12887_2020_2398_MOESM1_ESM.png]
